# Fibroadenoma: to biopsy or not to biopsy? That is the question

**DOI:** 10.1186/bcr3299

**Published:** 2012-11-09

**Authors:** AS Karuppiah, WG Bugg, A Juette

**Affiliations:** 1 Norfolk and Norwich University Hospitals NHS Trust, Norwich, UK

## Introduction

In 2011, our institution adopted the policy (in line with national guidance) not to biopsy breast masses in patients <25 years if they have benign characteristics on ultrasound. This retrospective audit was performed prior to this decision. We compare the histology result with the USS findings of all patients <25 years who had a breast mass biopsied or excised, between 2003 and 2009, with respect to Stavros criteria.

## Methods

Cases were identified from the Histopathology databases. Inclusion criteria include patients <25 years with a breast core biopsy or excision specimen. The ultrasound images were reviewed blind by a consultant breast radiologist and two registrars using Stavros criteria. The team knew one case; a blinded opinion from other consultant breast radiologists was sought.

## Results

The cohort included 109 cases, of which two were malignant, three were benign phyllodes and 104 were fibroadenomata. Twenty-three out of 109 cases did not meet Stavros criteria for a benign lesion, which included one malignant spindle cell tumour, one phyllodes and 21 fibroadenomata. The one case known to the team was reviewed blind by a consultant survey and the consensus opinion was that this did not meet Stavros criteria. Eighty-five out of 109 cases met Stavros criteria for a benign lesion. Of these, 52 cases had a core biopsy and 33 cases had a surgical excision biopsy.

## Conclusion

All nonbenign cases were correctly identified by all reviewers using Stavros criteria. No malignancies were missed in this series. This audit suggests that the biopsy rate can be safely reduced in those <25 years, if the national guidance is followed.

